# The Treatment of Typhoid Fever

**Published:** 1898-10

**Authors:** J. Sanderson Christison

**Affiliations:** Chicago


### The Treatment of Typhoid Fever.

CHICAGO, September 10, 1898.

TO THE EDITOR:—While much, of late, has been written on the treatment of typhoid fever, I feel that a brief statement of my experience may be of some value to others, especially as I seem to have met with greater success than any one else whose reports I have read. I have thus far hesitated to relate my experience, because of the fact that when I changed my location, several years ago, to adopt a more exclusive practice, I destroyed some of my records. However, I can recall at least twenty-eight cases in my last four years of general practice, and I think there were more in that period. But one can easier recall deaths than cases. Two deaths occurred among my typhoid fever cases during the four years in question, but one of these two cases was moribund when I first saw it. It was the case of a young man brought home from the pineries, suffering with the fever. When I first saw him he was delirious, and he died two or three days later. The other case was a middle-aged man who was allowed to get out of bed frequently, but contrary to my instructions, and suddenly expired, although his condition had not been particularly alarming. No post-mortem was held.

One of my cases of typhoid was a robust farmer, who recovered, but prematurely left home for a week to attend to some business. He returned sick, having a premonition of impending death, with what I took to be diphtheria. He died in a few days. Post-mortem revealed the intestines normal, but kidneys greatly congested. Thus it will appear that in reality I only had one death among the cases which I treated from start to finish.

My treatment had special reference to the following dangers: loss of vitality; germ breeding in the intestines; perforation and hemorrhage.

The first evil was met by isolation in as quiet a room as accommodations afforded. I strictly prohibited visiting by friends on penalty of my quitting the case. Relatives were allowed to visit for a few moments during certain hours of the day. I often had much fighting to do on this point, but I pushed my demand with severity, and invariably won respect in the end. A glassful of milk was given punctually every four hours, supped from a spoon. When half the glassful was taken, a few minutes' rest was given before the remainder was supped. Sometimes a dose of lactopeptin or liq. pancreatin followed the milk. Grapes, if procurable, were allowed freely, as was also cold water to drink in small quantities at a time. Every four or six hours, and sometimes oftener, the entire body was gently wiped with a soft piece of muslin dipped in lukewarm water containing a teaspoonful of bicarbonate of soda to the pint. The wiping and drying was done in patches, and the proceeding was not only gently but patiently accomplished. This process of cleansing and cooling had the effect of mild massage, and was invariably refreshing if done *gently* and *slowly*. Frontal headache was usually relieved by dipping a piece of muslin in a solution of cyanid of potash in laurel water (10 grains to the ounce) and laying it on the forehead for ten or thirty minutes.

The second point was supposed to be met by increasing the flow of bile into the intestine with one to three grains of calomel twice a day. Codein was given if griping was experienced. The calomel was kept up until the crisis was over.

The third danger seemed to be met by giving three or four drops of rectified turpentine every four hours, to diminish the gas in the inflated bowel and thus reduce the liability to perforation from distention of the bowel and the pressure of the gas on its ulcerated surfaces. Kidney complication was watched for, but the turpentine was seldom suspended if the bowel was markedly tympanitic.

The fourth point—hemorrhage—seemed to be forestalled by giving a dram, more or less, of the fluid extract of hamamelis every four hours. About twelve years ago I read an article in the *British Medical Journal*, by Shoemaker, of Philadelphia, on "Hemamelis as a Vascular Constringent, etc." I then began to use it in a variety of varicose and hemorrhagic cases, and was so favorably impressed with it that I concluded that its constringent, astringent and tonic properties would be serviceable in typhoid fever, as a preventive of hemorrhage, if nothing more. I therefore adopted the following prescription, which was only modified to meet the factors of the personal equation:

℞ Spts. terebinth. rect..............................ʒi<br>

Spts. juniper.......................... ......ʒss<br>

Fld. ex. hamamelis (P. D. & Co.).......℥ii<br>

P. acacia..........................................℥iss<br>

Aq. qs. ad............................ ........℥vi

Sig.: Dessertspoonful every four hours while awake.

All the cases ran a moderate course and seemed to be abbreviated, as compared with my experience for ten years prior. The diarrhœa seldom called for interference. Medicine and milk alternated every two hours, but were, as a rule, not allowed to interrupt sleep for two hours more if sleep continued that long. The cases were mostly sporadic and mostly in the country. Several families had three or four cases at the same time. A few were fat, flabby women of very low physiologic tone.—J. Sanderson Christison, in *Journal of the American Medical Association*.

